# Mixed model performance contracting and casemix in limited settings – the case of Lebanon

**DOI:** 10.1186/1472-6963-15-S2-A5

**Published:** 2015-06-15

**Authors:** Jade Khalife, Walid Ammar, Jihad Makouk, Rita Freiha, Hilda Harb, Fadi El-Jardali

**Affiliations:** 1 Ministry of Public Health, Beirut, Lebanon; 2 Faculty of Health Sciences, American University of Beirut, Beirut, Lebanon

## Background

Lebanon is an upper-middle income country with 4.5 million inhabitants (as well as more than 1.2 million refugees) and total health expenditures at 7.3% of the Gross Domestic Product. Lebanon has a mixed public and private healthcare sector. The Ministry of Public Health (MoPH) functions as ‘insurer of last resort’ for approximately 54% of the population that would otherwise lack hospitalization coverage. The MoPH contracts 26 public and 105 private hospitals and covers about 240,000 hospital admissions annually.

Since 2001, hospital contracting is based on a link between reimbursement rate and accreditation. Accreditation has encouraged the development of a healthcare quality culture among providers, and has likely contributed to total quality management. Yet, the sole link between accreditation and reimbursement has imposed limitations that are apparent to both MoPH and the hospitals. To address this problem, since 2009, a MoPH cross-collaboration team (with membership that includes implementers, policymakers, and researchers) has worked to develop a system to capture additional dimensions of healthcare performance.

## Materials and methods

We examined the appropriateness of linking hospital accreditation to reimbursement. Yang and Reinke (2006) suggest that, in the absence of national DRGs, an ICD-based casemix index (CMI) may be used, and we adopted this approach. We used the CMI to assess 122 hospitals for findings based on accreditation, ownership, and size. Findings indicate that only the highest-accredited (and reimbursed) hospital category had a high CMI, and that there is significant variation within accreditation categories, which suggests the presence of unfairness and inefficiencies.

Following dissemination of our findings and discussion with stakeholders, we used hospitalization data for the most recent year (2012-13) to calculate CMI for all surgical and 2-15 day medical hospitalizations (using ICD coding for medical cases, and CPT coding for surgical cases). The dataset included about 76% of the MoPH-covered admissions that occurred between June 2012 and May 2013.

Contracting scores were developed for each hospital based on the following factors and weights: a. 2012 accreditation results (40%); b. Casemix index (35%); c. Patient satisfaction (10%); d. ICU patient proportion (5%); e. Surgical-to-medical patient proportion (5%); and f. MoPH deduction for inappropriate billing (5%). The goal was to create a pricing system that reflects service complexity and quality, incentivizes good hospital practices, and discourages abuse. We calculated contracting score mean and standard deviation. Hospitals with z-score of 0 or above had the highest tariff (T1); those with z-scores between 0 and -0.5 had the middle tariff (T2); and those with z-scores below -0.5 had the lowest tariff (T3).

## Results

Among private hospitals 29 were T1, 44 were T2, and 33 were T3. Among public hospitals 9 were T1, 6 were T2, and 9 were T3. Results were discussed by policymakers from MoPH and the Syndicate of Private Hospitals; implementation of the updated tariffs occurred in November 2014.

## Conclusion

The new system, a ‘mixed-model’ for performance contracting, incorporates accreditation, casemix, patient satisfaction, and policy-oriented indicators. Additional indicators on patient outcomes are expected to be included in the upcoming 2015 contracting round. Evidence on the impact of pay-for-performance suggests early gains in several countries, but these changes may not be sustained over the long term. A locally tailored approach, which is updated and informed by policymakers and recent evidence, is critical to improve patient outcomes and resource allocation efficiency — particularly in limited-setting countries like Lebanon.

